# Cultural Models of *Well-Being* Implicit in Four Ghanaian Languages

**DOI:** 10.3389/fpsyg.2020.01798

**Published:** 2020-07-28

**Authors:** Annabella Osei-Tutu, Vivian A. Dzokoto, Adjeiwa Akosua Affram, Glenn Adams, Joakim Norberg, Bertjan Doosje

**Affiliations:** ^1^Goethe University Frankfurt, Post-Doctoral Fellowship-Programme, Frankfurt, Germany; ^2^Department of Psychology, University of Ghana, Accra, Ghana; ^3^Department of African American Studies, Virginia Commonwealth University, Richmond, VA, United States; ^4^Department of Psychology, The University of Kansas, Lawrence, KS, United States; ^5^School of Law, Psychology and Social Work, Örebro University, Örebro, Sweden; ^6^Social Psychology, University of Amsterdam, Amsterdam, Netherlands

**Keywords:** African cultural models, well-being, peace of mind, good living, relationality, affective states

## Abstract

This contribution to the collection of articles on “African Cultural Models” considers the topic of well-being. Reflecting modern individualist selfways of North American and European worlds, normative conceptions of well-being in hegemonic psychological science tend to valorize self-acceptance, personal growth, and autonomy. In contrast, given the embedded interdependence of everyday life in many West African worlds, one can hypothesize that cultural models of well-being in many Ghanaian settings will place greater emphasis on sustainability-oriented themes of material sufficiency and successful navigation of normative obligations. To explore this hypothesis, we interviewed local cultural experts who function as custodians of religion and an important source of support for well-being in many Ghanaian settings. We asked participants to identify and explain models of well-being implicit in four Ghanaian languages (Akan, Dagbani, Ewe, and Ga). Participants were 19 men and 15 women (age range 32–92 years; Mean = 59.83; SD: 14.01). Results reveal some features of local models, including *good health* and *positive affective states*, that appear to resonate with standard understandings of well-being in hegemonic psychological science. However, results also provide evidence for other features of local models – specifically, good living (including *moral living*, *material success*, and *proper relationality*) and *peace of mind* – associated with a sustainability or maintenance orientation to well-being.

## Introduction

Well-being is a quintessentially psychological topic, evident in enduring concerns with adjustment and optimal functioning. Recently, well-being has also become an increasingly important topic in sociology, political science, and economics as national governments and international development organizations have made well-being (rather than economic development *per se*) a central policy goal (White, 2010, 2015, 2017; Diener and Tay, 2015). “Good health and well-being” is third (after “no poverty” and “zero hunger”) on the list of 17 United Nations Sustainable Development Goals (United Nations Organization, 2015). Nations as diverse as the United Kingdom (Office for National Statistics, 2019) and Bhutan (Ura et al., 2012) track national well-being and personal happiness of their respective countries. The United Arab Emirates (Taylor, 2016) and Venezuela (Nagel, 2013) have governmental ministries for happiness, and a Nigerian state recently made headlines after it appointed a commissioner of happiness (Amah and Uanikhehi, 2017).

The increased attention to well-being as an important policy outcome has prompted an increased focus on conception and measurement of well-being. As with many topics, researchers have proposed that standard conceptions and measures of well-being in hegemonic psychological science have their foundation in modern individualistic constructions of person and society associated with settings that are WEIRD – that is, Western, educated, industrialized, rich, and (supposedly) democratic (see Diener et al., 2003; Arku et al., 2008; Henrich et al., 2010). As a result, hegemonic conceptions of well-being “reflect largely Western and, possibly, middle- and upper class characterizations of what it means to live a full and satisfying life” (Keyes et al., 2002: pg. 1019).

### Conceptual Framework

An important conceptual framework from which to theorize cultural-ecological variation in well-being comes from the influential work of Hazel Markus and Shinobu Kitayama (e.g., Markus and Kitayama, 1991, 1994, 2003, 2010; Markus et al., 1997). According to this framework, theory and research in hegemonic psychological science generally reflects (and portrays as “just natural”) historically particular constructions of self associated with modern individualism (e.g., Inkeles, 1969; Inkeles and Smith, 1974) of an increasingly neoliberal or post-materialist variety (Inglehart and Baker, 2000; Adams et al., 2019). Cultural ecologies of modern individualism – and the relational mobility associated with these cultural ecologies – afford an experience of self in terms of defining or essential attributes (e.g., traits, preferences, abilities) abstracted from particular contexts and relatively free from contextual constraints. They afford a *promotion-oriented* conception of well-being as personal fulfillment or self-expansion, in which a “full and satisfying life” involves exploration and expression of authentic interests and desires. The subjective experience of satisfaction looms large in this conception of well-being, as people exercise freedom of choice toward attractive options in pursuit of high-arousal positive affect (Tsai, 2007, 2017; Adams et al., 2019).

Standard conceptions of well-being resonate with the WEIRD minority of people in cultural ecologies of modern individualism. However, they resonate less well with the global majority of people across time and space who have inhabited cultural ecologies of embeddedness that afford a more “interdependent” (Markus and Kitayama, 1991) experience of self in terms of rootedness in place or inherent connection to social and physical context. Everyday life in these cultural ecologies affords a prevention-oriented conception of well-being that emphasizes fulfillment of obligations and careful management of relationships (Kitayama et al., 2010; Hitokoto and Uchida, 2015). The care associated with these selfways is not so much due to an inherently prosocial desire for harmony, but instead to a healthy appreciation for the destructive potential of conflict given realities of embeddedness. The subjective experience of satisfaction is less prominent (Kitayama et al., 2000; Shin et al., 2018); instead, conceptions of well-being emphasize low-arousal positive affective experiences of contentment, relief, or peace of mind as a signal that one has met social expectations (Kitayama et al., 2000; Suh and Oishi, 2002; Shin et al., 2018).

### The Present Study

As the pattern of citation in the previous section suggests, most research on cultural variation in conceptions of well-being has compared Euro-American and East Asian settings. The current study expands the focus of consideration to African settings. Cultural ecologies in many African settings promote relational ways of (well-)being that are attuned to the embedded interdependence of everyday life (e.g., Adams and Dzokoto, 2003). Yet, despite the prevalence of embedded interdependent selfways, researchers who investigate well-being in African settings typically impose hegemonic conceptions and measures that resonate with the modern individualism of WEIRD settings. The few studies that considered local, endogenous, or indigenous constructions of well-being in African settings have provided evidence consistent with the theoretical framework that we articulated earlier. Researchers in a Zambian community reported that, relative to hegemonic constructions of well-being, Chiawa constructions emphasized economic social (e.g., fulfilling obligations toward kin) and material (e.g., livelihood) dimensions but de-emphasized psychological dimensions (White and Jha, 2018). Researchers in another study reported that lay conceptions of well-being among an adult sample in Ghana had a multifaceted emphasis on social, economic, emotional/psychological, and physical health dimensions (Fadiji et al., 2019). However, because these researchers collected data in the medium of English using the English word for the concept (i.e., “well-being”), it is unclear whether the procedure influenced participant responses and experience toward WEIRD constructions of well-being (e.g., evidence of an emotional or psychological dimension).

The current study extends previous work in a direction consistent with the focus of this *Frontiers* collection on “African Cultural Models.” Rather than investigate conceptions and experience of well-being among students (e.g., Shin et al., 2018) or community samples (e.g., Delle Fave et al., 2016; Fadiji et al., 2019), we examine cultural models of well-being implicit in terminology of four Ghanaian languages. As we define the term, *cultural models* refer to systems of beliefs and values given material expression in the practices and artifacts of particular cultural worlds. Although the capacity for language may be a universal feature of human psychology, the languages that different societies use are particular cultural products (Gleason, 1961) that function as repositories of cultural models (Ameka, 2004; Kesebir and Kesebir, 2017; Dzokoto et al., 2018).

Because our focus was on cultural models rather than individual notions or idiosyncratic experience, we sought participants from a category of experts – custodians of religion – who could speak authoritatively about local constructions of well-being. Religion is an important source of cultural knowledge about appropriate aspirations, values, and ways of being for living a good life (Tsai, 2007; Joshanloo, 2019). This is perhaps especially true in West African settings like Ghana, where religion tends to permeate everyday life (Heaton et al., 2009), and over 95 percent of the population practice at least one of the three dominant religious traditions: Christianity, Islam, and African Traditional Religion (Ghana Statistical Service, 2012). Besides their role as moral guides, religious leaders in Ghana fill an important role as counselors who minister to the well-being of ordinary people. People often entrust their well-being into the hands of religious leaders who help them manage stressful problems and offer advice about socially appropriate actions (Osafo et al., 2015; Kpobi and Swartz, 2018a, b, 2019; Osei-Tutu et al., 2019).

The four languages that were the focus of our investigation are Akan, Dagbani, Ewe, and Ga. Akan is a collection of mutually intelligible Kwa languages associated primarily with regions of southern Ghana. Akan speakers constitute the biggest linguistic group in Ghana, with over 47% of the total population of 25 million persons (Ameka and Kropp Dakubu, 2008). Ewe is a Kwa language associated with regions in eastern Ghana; it is the primary language for approximately 13.9% of the Ghanaian population. Ga is a Kwa language associated with the Greater Accra Region of Ghana; it is the primary language for approximately 7.4% of the Ghanaian population (Ameka and Kropp Dakubu, 2008; Ghana Statistical Service, 2012). The most dissimilar of the four languages was Dagbani, a Gur language associated with regions in northern Ghana where Islamic traditions are relatively strong. It is the primary language for approximately 16% of the Ghanaian population (Ghana Statistical Service, 2012; Hudu, 2013).

Although our investigation was primarily exploratory in character, previous research on cultural variation in experience of well-being and the theoretical framework that we outlined in the introduction suggested a few hypotheses. The primary hypothesis (H1) was that conceptions of well-being implicit in local language terms would not show the emphasis on psychological fulfillment associated with growth-oriented conceptions of well-being in WEIRD settings and hegemonic psychological science. Instead, theory and research suggest the hypothesis (H2) that local conceptions of well-being implicit in the Ghanaian languages will emphasize themes – namely, social responsibility, fulfillment of interpersonal obligations, and a normative-contextual focus on material conditions rather than subjective judgments of emotional states (Suh and Oishi, 2002; Riemer et al., 2014) – associated with sustainability-oriented conceptions of well-being in cultural ecologies of embeddedness and interdependence. Similarly, in contrast to the high-arousal positive affect (e.g., excitement and happiness) associated with personal fulfillment, theory and research suggest the hypothesis (H3) that local conceptions of well-being implicit in the Ghanaian languages will emphasize the low-arousal positive affective experience (e.g., feelings of relief or peace of mind) associated with fulfillment of social expectations. Finally, research on somatization of affect and the prominence of body referents in Ghanaian-language emotion terms (Ameka, 2002; Dzokoto and Okazaki, 2006; Dzokoto, 2010; Dzokoto et al., 2016; Agyekum, 2019), as well as a normative-contextual focus on material conditions rather than emotional states, suggests the hypothesis (H4) that local language terms will emphasize physical health manifestations of well-being.

## Materials and Methods

### Participants

Participants were 19 men and 15 women, aged between 32 and 92 years (Mean = 59.83; SD: 14.01); selected from four regions of Ghana: Greater Accra, Central, Volta, and Northern. Participants included native speakers of Akan (*n* = 13); Dagbani (*n* = 7); Ewe (*n* = 6); and Ga (*n* = 8) languages. We interviewed Akan speakers in the Central and Greater Accra Region. We interviewed all Ewe speakers in the Volta Region and all Ga speakers in the Greater Accra Region. We interviewed the majority of Dagbani speakers (*n* = 6) in the Northern Region, but one in the Greater Accra Region. Participants were custodians of religion including African Traditional Religion (*n* = 18); Islam (*n* = 11); and Christianity (*n* = 5).

### Materials

Prior to starting fieldwork, the researchers consulted linguists and translators of the four languages to develop the data-collection guide. The consultants assisted in identifying possible local concepts that the research team could use as prompt words to initiate the conversation around well-being with prospective participants. All the four languages in this study can accommodate direct translations of the concept of well-being. However, such translations may be inadequate to capture the richness of the meanings of well-being. For example, in Akan “well” translates into *pa* and “being” translates into *asetena* or *abrabɔ*. Accordingly, the research team could have used the direct translation *asetenap*a or *abrabɔpa*. However, the direct translation did not fully capture the richness of the concept of well-being in Akan. Since the focus of this study was not to look for linguistic equivalents, but to find local understandings of well-being, we engaged with the consultants to assist in identifying broad prompt words in the local languages that we could use to initiate conversations and elicit concepts of well-being in the respective languages. Based on the feedback from the consultants, we identified *Yi ye di* [living well] as well as *asetena mu ye* [life is going well for someone] as prompt words for Akan. We used *biεhisuŋ* [good/decent living] as a prompt for the Dagbani interviews. In Ewe, we identified *agbe nyue nɔ nɔ* [good living/living well]. The prompt words for Ga were *miishε* [happiness] and *shihilε kpakpa* [good life]. The list of prompt words grew with every interview as participants provided alternative concepts.

### Procedure

Before collecting data, we obtained ethical certification from the Ethics Committee for the Humanities, University of Ghana. We collected data between January and June 2019. Interviewers included two men and four women with bachelors or postgraduate degrees in the social sciences. Native speakers conducted the interviews.

Interviewers approached prospective participants at their homes and work locations (including shrines, churches, and secular workplaces). For some participants, researchers used pre-existing research contacts. Visiting homes for research purposes is not unique to our study. The communal nature of Ghanaian settings permits researchers to approach participants in their homes (see Osei-Tutu et al., 2018a), and the practice is culturally acceptable or even preferable. On visiting prospective participants, interviewers explained the purpose of the visit: that is, to learn about ideas related to well-being in the local language of the prospective participant. If the person agreed, the interviewers discussed a suitable time for the interview. Prior to the interview, the participant completed a verbal consent procedure. The interviewer then conducted one-on-one interviews to elicit concepts related to well-being in the participant’s native language. For participants who were bilingual speakers (of English and their native language) the interviewer began the interview as follows:

*We will be talking about what the English term as* well-being. *I will say well-being comprises of so many things like everything moving on well with your life. Please, do you think you can get one word to represent this well-being I am talking about in your language?*

For participants who were monolingual speakers of their native language, the interviewer began each interview by asking (in the appropriate language), *“*Are there any concepts in [insert language] that relate to the concept of [insert prompt word]?” In all cases, the interviewer asked follow-up questions (e.g., “What other concept can be used to describe [insert given local word]?”) to elicit a broad range of responses. The interviews continued until participants indicated that they had exhausted all words they could relate to well-being.

Five assistants with competence in both English and one of the local languages transcribed the interviews. Interviewers for the Ga and Dagbani sessions transcribed the resulting interviews. One interviewer and another assistant transcribed the Akan interviews. An assistant transcribed the Ewe interviews. All assistants followed the same transcription guidelines. They simultaneously translated and transcribed the interviews into English with the exception of local well-being concepts, which they transcribed in untranslated form with the English translation in parenthesis. The first author supervised the transcription and where necessary consulted linguists about responses that were ambiguous or otherwise difficult to translate.

We analyzed responses via an inductive process with multiple iterations. In the initial step, the third author created a coding frame using Microsoft Excel. The frame comprised the prompt words, the local concept(s) elicited from the interviews, and a summary of the meanings of well-being for each participant. Examples appear in Table 1.

**TABLE 1:** Layout of initial coding of well-being terms.

Transcript No.	Well-being word prompt	Local word response	Corresponding meaning
C4	*Asetenamu ye* [Akan]	1. *ahotɔ* 2. *asomdwe* 3. *anigye* 4. *nokwaredi*	1. without worries 2. peace of mind 3. happiness 4. truthfulness
T5	*biεhisuŋ* [Dagbani]	1). *suγulo* 2). *viεnyεliŋga* 3). *suhupiεlli*	1). patience 2). living properly/decently/well 3). happiness
E1	*Agbe nyue nɔ nɔ* [Ewe]	1. *nɔnɔme nyo ne* 2. *agbe nɔ nɔ nyue*	1. living in a better condition 2. living in the right character
AP5	*Well-being^1^* [Ga participant]	1).*miishεε* 2). *hedzɔlε* 3). *shihilε kpakpa* 4). *hewalε na mɔ* 5). *ni okε onyemi ten ye ekpakpa* 6). *ekomefeimɔ fεε mɔ*	1). happiness 2). peace of mind 3). good livelihood/good life 4). having good health 5). good relationship with your friends or people around 6). working in unity

*^1^For some bilingual speakers, we gave the prompt word in English and they responded in their native language.*

Next, a team comprising the first, third, and fourth authors met on multiple occasions to review the coding frame. The team first looked for meanings of the local concepts as presented in the transcripts. The team then examined and identified concepts that were frequent within and across each of the four languages. We grouped similar terms together both within and between languages. As an example within languages, we combined Ewe terms *nutifafa* [peace; literally, “body-red-become-cool”] and *tomefafa* [peace; literally, “ear-inside-red-become-cool”] under the category related to “peace” (F. K. Ameka, personal communication, December 10, 2018). As an example across languages, we combined Akan *asomdwe* [peace of mind], Ga *toijorlε* [peace of mind], and Ewe *nutifafa* [peace] under “peace of mind” (Table 2).

**TABLE 2:** Common Ghanaian understanding of wellbeing.

Theme	Local language lexica			
	
	Akan	Dagbani	Ga	Ewe
Good living	• *asetenapa/abrabɔpa* • [good life] • *asetenamu ye* [life well for someone] • *yiyeyε* [success] • *yiyedi/ate yiye* [living well] •*ɔdeneho* [self-sufficient]	• *biεhisuŋ* [decent living/character] • *halisuŋ* [good character] • *naŋgbanyini* [unity] • *suγulo* [patience]*• viεnyεliŋga* [decent/proper living] • *yurilim* [love] • *ʒisuŋ* [good/comfort/standard of living] • *yεlimaŋli* [good speech/truth]	• *nɔ yaa* [progress/development]*•shihilε kpakpa* [good living] • *ehi ehabo* [things are going well for you] • *shweremɔ* [flourishing] *bulε* [respect]	• *de di nɔnɔ*/*de di nyenye* [well-being] • *agbe me nyo na amide* [life is well for someone] • *agbe nyue nɔnɔ* •[good living] • *dzidzeme* [fulfilled] • *sugoso* [self-sufficient]
Good health		• *alaafee* [good health/wellness/welfare]	• *gbɔmɔtso hewalε* •[healthy body/living]	• *lamase* [good health]
Positive affective state	• *anigye* [happiness]	• *suhupiεlli* [happiness]	• *miishεε* [happiness]	• *dzi-dzɔ* [joy]
Peace of mind	• *ahotɔ* •[without worries] • *asomdwe* •[peace of mind]		• *toijorlε* [peace of mind] • *tsuimlijorlε* [peace of mind] •*hejorlε* [peace]	• *nutifafa* [peace] • *tomefafa* [peace]

Finally, for validation purposes, another team comprising the first, second, and fourth authors reviewed the findings and presented them at two separate academic events to audiences of linguists and experts in the four local languages. We used feedback from the presentations to refine the results that we present in this paper. For example, after a seminar presentation of preliminary results, the authors received feedback that our procedure did not capture some Dagbani words associated with well-being. In response, the researchers conducted additional interviews to elicit additional Dagbani concepts. Review of results by peers with thorough cultural understanding of Ghanaian languages and context served to enhance the trustworthiness of the findings (Morrow, 2005), which in qualitative research is analogous to reliability and credibility in quantitative research (Lincoln and Guba, 1986).

## Results

Four major themes were evident in the words that participants generated in response to our prompts. These themes and associated words appear in Table 2. Our focus was on cultural models implicit in the words and their meanings, rather than participant understandings of well-being, *per se*. However, we quote participants at length to illuminate the meanings that they ascribed to the words that they nominated.

### Good Living

A major theme and largest category consisted of words related to “good living” or other more-or-less direct translations of the English *well-being*: Akan *abrabɔpa*, Ewe *de di nɔ nɔ*, Ga *shihilε kpakpa*, and Dagbani *ʒisuŋ*. The roots of each word – *abrabɔ, de di, shihilε*, and *ʒi* – translate as “life.” The suffixes – *-pa*, -*nɔ nɔ*, -*kpakpa*, and -*suŋ* – loosely translate as “good.” Of course, the concept of “good living” is ambiguous, with many possible meanings that it is the goal of this project to chart.

#### Morality

One way to disambiguate meaning is to refer to participants’ explanation of words for “good living” in the context of the interviews.

[E6]: *For* de di nɔnɔ, *in this life, that one shows that, we would just say that, it’s coming from heaven, or that person, he would lack nothing. Err, he obeys the laws, and does everything, he has money, and he doesn’t want trouble, err, he is like an angel (God’s messenger) that came to this earth. He doesn’t need anything. When someone is in trouble, he takes the person out of it, and he does a lot of things, which makes us to call them, or see them as a person who is well* (Ewe, male, African Traditional Religion).

[G6]: Shihilε kpakpa *[good living] is doing something good for someone* (Ga, female, African Traditional Religion).

The context suggests that many participants understood words for “good living” to refer to a morally upright way of life. The implication is that moral virtue is productive not only of spiritual or social health, but also physical health.

#### Material Success

Another way to disambiguate understandings of “good living” comes from other words people gave in response to prompts that were not direct translations of *well-being*. One set of words under the “good living” theme related to material success. Participants in three languages mentioned words (*asetenamu ye* [Akan]; and *agbe me nyo na amide* [Ewe]; and *ehi ehabo* [Ga]) that translate as “life is well for someone.” This phrase suggests an observer’s assessment of the material conditions of another person’s life, a construction that contrasts directly with the emphasis in hegemonic psychological science on subjective judgments of well-being (Camfield and McGregor, 2005; Oishi et al., 2013; Suh and Choi, 2018).

The emphasis on material success was likewise evident in the Akan *yiyeyε* [success], *ate yiye* [good living], *yiyedi* [living well], and *ɔdeneho* [self-sufficient]; Ga *nɔyaa* [progress/development] and *shweremɔ* [flourishing]; and Ewe *sugoso* [self-sufficient] and *dzidzeme* [fulfilled]. At first glance, the English translation of these words might lead one to conclude that they map the same concept space of psychological growth and fulfilment associated with conceptions of well-being in hegemonic psychological science. However, the context of the interview implies a focus on the objective material conditions of life – success at achieving a level of comfort and material security (see Osei-Tutu et al., 2018b) – rather than a subjective sense of psychological well-being.

[C1]: Woa ye yei, *the stature now means I have a post, having a post [social rank] like I have my own skills, I used to work and I have my own inheritance. I have my own money, I’m wealthy* (Akan, female, African Traditional Religion).

[AP2]: Shihilε kpakpa, *yes, it means everything is going well with you. That is life, your life is going well. And like I said, when they say that, they are basically thinking of food, shelter and clothing. That is what they mean by* shihilε kpakpa. *So when they are dressing, you can also dress. When it is good food, you can also eat. When it is somewhere to stay, sleep or watch your TV comfortably, you also have* (Ga, female, Christian).

[E1]: Sugoso, *that one means that the person needs nothing in life [*…*] he has properties; he can’t ask and receive anything from anyone because he has everything* (Ewe, male, African Traditional Religion).

[E3]: …*when they say “*agbe dzedzi na ame” *it means, the person is in the world, and he sees the successful end of everything he does. He gets food to eat, gets cloth to put on; he gets work to do; then it shows that, life has fallen in place for him* (Ewe, male, African Traditional Religion).

Consistent with hypothesis (H1), interviews provided little evidence for explicit reference to promotion-oriented themes of psychological fulfilment in cultural models of well-being. In contrast, and consistent with hypothesis (H2), results indicated evidence for an emphasis on material conditions of life.

#### Proper Relationality

Similarly consistent with hypothesis (H2), results also indicated evidence in cultural models of well-being for prevention-oriented ideas of social responsibility. These ideas were evident in a second set of words under the “good living” theme related to proper relationality.

[T6]: *It is a kind of person who has* suγulo *[patience] and* viεnyεliŋga *[decent/proper living] with his or her fellow human being* (Dagbani, male, Muslim).

[G4]: *Another concept is* bulε *[respect].* Shihilε kpakpa *[good living] is respect. If you live with someone and you respect him or her and do the right thing it is a sign of good living*… (Ga, female, African Traditional Religion).

[T4]: *When we say a person or someone has* biεhisuŋ *he or she lives in harmony or has good moral values among the colleagues. He or she has* halisuŋ *[good character/conduct]* (Dagbani, female, Muslim).

Our decision to categorize these ideas under the theme of “good living” reflects the polysemy of the Ghanaian language phrases that serve as translations of *well-being*. For example, depending on the context, *asetenapa* can refer to proper sociality, material success, or morally appropriate living. In one context, Akan speakers might describe a person enjoying a high standard of living as living *asetenapa*. In another context, Akan speakers might describe a person who does not have social disruptions in their familial relationships and who has fulfilled their obligations toward kin as leading *asetenapa* (see, Van der Geest, 1998; Osei-Tutu et al., 2018b). In yet another context, Akan speakers might describe a person who is perceived to be leading a morally upright life as leading *asetenapa.* Our focus in the current work was to elicit words that speakers of Ghanaian languages use for the concept of *well-being*. The task of disambiguating the different senses of words that translate as “good living” remains a topic for future research.

### Good Health

A second theme that was evident in the words that participants generated in response to interview prompts is that of *good health*. This theme was explicitly evident across three of the languages, specifically in the Dagbani *alaafee*, Ewe *lamase*, and Ga *gbɔmɔtso hewalε.*

[E6]: Lamase; *When you wake up, you wake up healed. You don’t complain about being sick, and you see that, your blood vein and every other thing is working perfectly, you wake up healed and walk out of the room, God helps you, and you get good health, you go to the market, go to farm, and you look for food to eat* (Ewe, male, African Traditional Religion).

Consistent with hypothesis (H4) and the emphasis on material success that we discussed in the previous section, this theme suggests a focus on objective conditions of a person’s life – especially freedom from disease and debility – rather than a subjective assessment of broader psychological fulfillment.

### Positive Affect

References to a more subjective experience of well-being were evident in a third theme that emerged from words that participants generated in response to interview prompts. Similar to hegemonic conceptions of well-being, participants from each language group responded with words – specifically, Akan *anigye*, Dagbani *suhupiεlli*, Ewe *dzidzɔ*, and Ga *miishεε* – that bilingual speakers typically translate as “happiness.” Despite the apparent similarity with hegemonic conceptions of well-being, it remains unclear whether these Ghanaian language words refer to the same high-arousal positive affective experience of euphoria and excitement typically associated with *happiness* in hegemonic psychology.

One clear point of divergence from hegemonic conceptions concerns the presence of body referents in these emotion words. Although speakers translate them into English as *happiness*, the literal translation of these words refers to bodily experience. The literal translation of Akan *anigye* is “eyes-have-received.” The literal translation of Dagbani *suhupiεlli* is “white heart.” The literal translation of Ewe *dzidzɔ* is “heart straight.” The literal translation of Ga *miishεε* is “body-full.” Here again, and consistent with hypothesis (H4), the expression of emotional experience in terms of tangible bodily experience implies a conception of well-being in terms of objective (bodily) conditions rather than subjective psychological assessment.

### Peace of Mind

Although words that translate as “happiness” may appear to resonate with hegemonic understandings of well-being, a fourth theme that emerged from responses to interview prompts was an emphasis on “peace of mind” that does not resonate with hegemonic conceptions. This theme was evident across three of the four languages: Akan *ahotɔ* [undisturbed/without worries] and *asomdwe* [peace of mind], Ewe *nutifafa* [peace] and *tomefafa* [peace], and Ga *toijorlε* [peace of mind] and *tsuimlijorlε* [peace of mind].

[AP6]: Ahotɔ *is you not thinking about anything, you are comfortable, you don’t have any sleepless things that pull you into pain* (Akan, male, Christian).

[G4]: Toijorlε *is for example in some family, there is understanding of each other. If there is any misunderstanding and someone walks in to settle it, the problem is settled there and then. So if you come from such a family and you are not around, you can never be troubled because you know there’s peace, and that is what I mean by good living or peace of mind or wellbeing* (Ga, female, African Traditional Religion).

The literal translations of these words – *ahotɔ* [body-cool], *asomdwe* [ear-cool], *nutifafa* [body-red-become-cool], *tomefafa* [ear-inside-red-become-cool], *toijorlε [*ear-cool*]*, and *tsuimlijorlε* [heart-inside-become-cool] – indicate somatic reference to a sensation of coolness that, in the hot climate, has connotations of relief (see Ameka, 2015, on temperature expressions in Ewe). Consistent with hypothesis H3, these words suggest an understanding of well-being in terms of low-arousal positive affective states related to contentment, calmness, serenity, and relief that one has met social expectations.

## General Discussion

We conducted this study to better understand cultural models of well-being in four major Ghanaian languages. Results suggest that well-being in these languages involves *good living* (including *moral living*, *material success*, and *proper relationality*), *good health, positive affective states*, and *peace of mind*. At first glance, some aspects of these constructions resonate with conventional understandings of well-being in hegemonic psychological science. For example, participants nominated concepts that translate in English as *happiness* or *fulfilment*. However, participant explanations give reason to doubt that experiences associated with these concepts are identical to those in hegemonic psychological science; specifically, Ghanaian concepts imply a more materially focused or embodied form of happiness and fulfilment. In this way, results resonate with research in cultural psychology, which suggests that different cultural settings afford different modes of happiness (Uchida and Kitayama, 2009; Hitokoto and Uchida, 2015). More generally, results did not provide much evidence for the emphasis on psychological fulfillment associated with standard, growth-oriented conceptions of well-being in hegemonic psychological science. Instead, interview responses emphasized themes of social responsibility, materiality (Coe, 2011), and low arousal positive affect (e.g., peace of mind; Tsai, 2007, 2017) that our theoretical framework associates with sustainability or maintenance-oriented conceptions of well-being in cultural ecologies of embeddedness and interdependence (Adams and Dzokoto, 2003).

Results are similar to those of a previous investigation that considered conceptions of well-being among urban-dwelling Ghanaian participants in the context of an interview in English (Fadiji et al., 2019). One point of divergence is the emphasis on moral living as a component of well-being. Although researchers in the previous study did not report an emphasis on moral living, participants in the current work did so in their elaboration on words that translate as “good living.” This emphasis on moral living resonates with the hypothesized prevention-oriented focus on proper relationality and dutiful attention to obligation in cultural ecologies of embeddedness (Higgins, 1996, 1997, 1998). In many Ghanaian (and other West African) communities, moral living has a strong relational component; a moral person is one who honors social obligations. However, it is also possible that the emphasis on this feature in the current study is an “artifact” of our sample of religious leaders, who may be especially likely to propose a construction of well-being as in terms of moral living. This remains a topic for future research.

### Limitations

Like any study, this investigation is not without limitations. The words that we elicited are not exhaustive of all words related to cultural models of well-being in the respective languages. For example, Dagbani speakers did not nominate words associated with the theme of *peace of mind* even though the concept exists in that language. It is possible that other approaches or interviews with people from other language groups or from other segments of the Ghanaian society may yield additional words. Another limitation is that the purpose of the study was to discern cultural models of well-being, but results do not tell us about the prominence of different models or their component themes. Similarly, it is not clear how different cultural models of well-being manifest in language map onto individual subjective experience of well-being. Finally, because our sample size was small and language categories were confounded with religious categories (e.g., all Dagbani speakers were Muslim), the design of the current research does not permit us to make conclusions about differences in cultural models across languages, as a function of religious tradition, or along other dimensions of sociocultural variation.

Accordingly, an important direction for future research is qualitative (Delle Fave et al., 2016) or quantitative (e.g., the GRID project; Fontaine et al., 2013) approaches to compare people’s subjective definitions or experience of well-being across language groups, religious traditions, and other forms of sociocultural engagement. Among other points, such research will help to illuminate whether the emphasis on peace of mind and de-emphasis of psychological fulfillment in Ghanaian language concepts corresponds to their relative prominence (or lack thereof) in everyday experience.

## Conclusion: Decolonial Caution About the Science of Well-Being

If the relative de-emphasis on psychological fulfillment or high-arousal positive affect does extend to individual experience, then should practitioners take this as a sign of suboptimal well-being? This is not simply an academic question; instead, the answer matters greatly for governments as they shape policy and devote scarce resources to assess and improve well-being. If conceptions of well-being differ across cultural settings, then whose conceptions should inform standard measures? Given global power dynamics, international institutions typically adopt conceptions, measures, and interventions that resonate with the modern individualist constructions of well-being that inform hegemonic psychological science (Adams et al., 2015; Bhatia and Priya, 2018; Oppong, 2019; Chaudhary and Sriram, 2020). What violence happens when international institutions impose these conceptions, measures, and interventions in cultural settings where they do not resonate. Do hegemonic conceptions and measures unfairly pathologize ways of (well-)being that have developed in attunement with cultural ecologies of embeddedness and interdependence? Would these settings – and perhaps people in general across cultural settings – be better served by conceptions and measures of well-being that better reflect realities of embeddedness and interdependence? Research on such questions is only beginning (Hitokoto and Uchida, 2015; White, 2017; Rappleye et al., 2020). We offer this work as a step in that direction.

## Data Availability Statement

The datasets generated for this study are available on request to the corresponding author.

## Ethics Statement

The studies involving human participants were reviewed and approved by the Ethics Committee of the Humanities, University of Ghana. Written informed consent for participation was not required for this study in accordance with the national legislation and the institutional requirements.

## Author Contributions

All authors contributed to the conception of the study. VD, AA, and AO-T contributed to the acquisition of the data. GA, AO-T, AA, and VD contributed to the analysis and interpretation of the data. AO-T contributed to the initial draft. GA, AA, and VD contributed to the revision and the final draft.

## Conflict of Interest

The authors declare that the research was conducted in the absence of any commercial or financial relationships that could be construed as a potential conflict of interest.

## References

[B1] Adams G. Dobles I. Gómez L. Kurtiş T. Molina L. E. (2015). Decolonizing psychological science: introduction to the special thematic session. *J. Soc. Polit. Psychol.* 3 213–238. 10.5964/jspp.v3i1.564

[B2] Adams G. Dzokoto V. A. (2003). Self and identity in African studies. *Self Identity* 2 345–359. 10.1080/714050252

[B3] Adams G. Estrada-Villalta S. Sullivan D. Markus H. R. (2019). The psychology of neoliberalism and the neoliberalism of psychology. *J. Soc. Issues* 75 189–216. 10.1111/josi.12305

[B4] Agyekum K. (2019). *Akan Body Part Expressions.* Accra: Adwinsa Publications (GH) Ltd.

[B5] Amah M. Uanikhehi I. (2017). *Not Everyone is Happy About This New ‘Happiness Minister’ in Nigeria.* Available online at: https://edition.cnn.com/2017/12/07/africa/nigerian-governor-appoints-happiness-minister/index.html (accessed March 4, 2020).

[B6] Ameka F. (2002). Cultural scripting of body parts for emotions: on ‘jealousy’ and related emotions in Ewe. *Pragmat. Cogn.* 10 27–55. 10.1075/pc.10.12.03ame

[B7] Ameka F. (2015). “Hard sun, hot weather, skin pain”: the cultural semantics of temperature expressions in Ewe and Likpe (West Africa),” in *Linguistics of Temperature*, ed. Koptjevskaja-Tamm M. (Amsterdam: John Benjamins Publishing Company), 43–72. 10.1075/tsl.107.02ame

[B8] Ameka F. K. (2004). Grammar and cultural practices: the grammaticalization of triadic communication in West African languages. *J. West Afr. Lang.* 30 5–28.

[B9] Ameka F. K. Kropp Dakubu M. E. (eds) (2008). *Aspect and Modality in Kwa Languages.* Amsterdam: John Benjamins Publishing Company.

[B10] Arku F. S. Filson G. C. Shute J. (2008). An empirical approach to the study of well-being among rural men and women in Ghana. *Soc. Indicat. Res.* 88 365–387. 10.1007/s11205-007-9197-0

[B11] Bhatia S. Priya K. R. (2018). Decolonizing culture: Euro-American psychology and the shaping of neoliberal selves in India. *Theory Psychol.* 28 645–668. 10.1177/0959354318791315

[B12] Camfield L. McGregor J. A. (2005). “Resilience and wellbeing in developing countries,” in *Handbook for Working with Children and Youth Pathways to Resilience Across Cultures and Contexts*, ed. Ungar M. (Thousand Oaks, CA: SAGE), 189–209.

[B13] Chaudhary N. Sriram S. (2020). Psychology in the “Backyards of the World”: experiences from India. *J. Cross Cult. Psychol.* 51 113–133. 10.1177/0022022119896652

[B14] Coe C. (2011). What is love? The materiality of care in Ghanaian transnational families. *Int. Migr.* 49 7–24. 10.1111/j.1468-2435.2011.00704.x 22180882

[B15] Delle Fave A. Brdar I. Wissing M. P. Araujo U. Castro Solano A. Freire T. (2016). Lay definitions of happiness across nations: the primacy of inner harmony and relational connectedness. *Front. Psychol.* 7:30. 10.3389/fpsyg.2016.00030 26858677 PMC4726797

[B16] Diener E. Oishi S. Lucas R. E. (2003). Personality, culture, and subjective well-being: emotional and cognitive evaluations of life. *Annu. Rev. Psychol.* 54 403–425. 10.1146/annurev.psych.54.101601.145056 12172000

[B17] Diener E. Tay L. (2015). Subjective well-being and human welfare around the world as reflected in the Gallup World Poll. *Int. J. Psychol.* 50 135–149. 10.1002/ijop.12136 25612012

[B18] Dzokoto V. (2010). Different ways of feeling: emotion and somatic awareness in Ghanaians and Euro-Americans. *J. Soc. Evol. Cult. Psychol.* 4 68–78. 10.1037/h0099299

[B19] Dzokoto V. Okazaki S. (2006). Happiness in the eye and heart: a comparison of somatic referencing in the emotion vocabularies of two West African emotion lexica. *J. Black Psychol.* 32 117–140. 10.1177/0095798406286799

[B20] Dzokoto V. Senft N. Kpobi L. Washington-Nortey P.-M. (2016). Their hands have lost their bones: exploring cultural scripts in two West African affect lexica. *J. Psycholinguist. Res.* 45 1473–1497. 10.1007/s10936-016-9415-5 26888786

[B21] Dzokoto V. A. Osei-Tutu A. Kyei J. J. Twum-Asante M. Attah D. A. Ahorsu D. K. (2018). Emotion norms, display rules, and regulation in the Akan society of Ghana: an exploration using proverbs. *Front. Psychol.* 9:1916. 10.3389/fpsyg.2018.01916 30429804 PMC6220724

[B22] Fadiji A. Meiring L. Wissing M. P. (2019). Understanding well-being in the Ghanaian context: linkages between lay conceptions of well-being and measures of hedonic and eudaimonic well-being. *Appl. Res. Qual. Life* 15:935 10.1007/s11482-019-09777-2

[B23] Fontaine J. Scherer K. Soriano C. (eds) (2013). *Components of Emotional Meaning: A Sourcebook.* Oxford: Oxford University Press.

[B24] Ghana Statistical Service (2012). *2010 Population and Housing Census: Summary Report of Final Results.* Accra: Sankofa Press Limited.

[B25] Gleason H. S. Jr. (1961). *An Introduction to Descriptive Linguistics.* New Delhi: Oxford and IBH Publishing Company.

[B26] Heaton T. James S. Ohene-Sakyi Y. (2009). Religion and socioeconomic attainment in Ghana. *Rev. Relig. Res.* 51 71–86.

[B27] Henrich J. Heine S. J. Norenzayan A. (2010). The weirdest people in the world? *Behav. Brain Sci.* 33 61–63. 20550733 10.1017/S0140525X0999152X

[B28] Higgins E. T. (1996). The “self digest”: self-knowledge serving self-regulatory functions. *J. Pers. Soc. Psychol.* 71 1062–1083. 10.1037/0022-3514.71.6.1062 8979379

[B29] Higgins E. T. (1997). Beyond pleasure and pain. *Am. Psychol.* 52 1280–1300. 10.1037/0003-066X.52.12.1280 9414606

[B30] Higgins E. T. (1998). “Promotion and prevention: regulatory focus as a motivational principle,” in *Advances in Experimental Social Psychology*, Vol. 30 ed. Zanna M. P. (New York, NY: Academic Press), 1–46. 10.1016/s0065-2601(08)60381-0

[B31] Hitokoto H. Uchida Y. (2015). Interdependent happiness: theoretical importance and measurement validity. *J. Happiness Stud.* 16 211–239. 10.1007/s10902-014-9505-8

[B32] Hudu F. (2013). Dagbani tongue-root harmony: triggers, targets and blockers. *J. Afr. Lang. Linguist.* 34 47–73. 10.1515/jall-2013-0002

[B33] Inglehart R. Baker W. E. (2000). Modernization, cultural change, and the persistence of traditional values. *Am. Sociol. Rev.* 65 19–51.

[B34] Inkeles A. (1969). Making men modern: on the causes and consequences of individual change in six developing countries. *Am. J. Sociol.* 75 208–255.

[B35] Inkeles A. Smith D. (1974). *Becoming Modern.* Cambridge, MA: Harvard University Press.

[B36] Joshanloo M. (2019). Cultural religiosity as the moderator of the relationship between affective experience and life satisfaction: a study in 147 countries. *Emotion* 19 629–636. 10.1037/emo0000469 29999383

[B37] Kesebir S. Kesebir P. (2017). A growing disconnection from nature is evident in cultural products. *Perspect. Psychol. Sci.* 12 258–269. 10.1177/1745691616662473 28346112

[B38] Keyes C. L. M. Smotkin D. Ryff C. D. (2002). Optimizing well-being: the empirical encounter of two traditions. *J. Pers. Soc. Psychol.* 82 1007–1022. 10.1037/0022-3514.82.6.1007 12051575

[B39] Kitayama S. Karasawa M. Curhan K. B. Ryff C. D. Markus H. R. (2010). Independence and interdependence predict health and wellbeing: divergent patterns in the United States and Japan. *Front. Psychology* 1:163. 10.3389/fpsyg.2010.00163 21833228 PMC3153777

[B40] Kitayama S. Markus H. R. Kurokawa S. (2000). Culture, emotion, and well-being: good feelings in Japan and the United States. *Cogn. Emot.* 14 93–124. 10.1080/026999300379003

[B41] Kpobi L. Swartz L. (2018a). ‘That is how the real mad people behave’: beliefs about and treatment of mental disorders by traditional medicine-men in Accra, Ghana. *Int. J. Soc. Psychiatry* 64 309–316. 10.1177/0020764018763705 29529921

[B42] Kpobi L. N. A. Swartz L. (2018b). ‘The threads in his mind have torn’: conceptualization and treatment of mental disorders by neo-prophetic Christian healers in Accra, Ghana. *Int. J. Ment. Health Syst.* 12:40. 10.1186/s13033-018-0222-2 30061921 PMC6056911

[B43] Kpobi L. N. A. Swartz L. (2019). Muslim traditional healers in Accra, Ghana: beliefs about and treatment of mental disorders. *J. Relig. Health* 58 833–846. 10.1007/s10943-018-0668-1 29992474

[B44] Lincoln Y. S. Guba E. G. (1986). “But is it rigorous? Trustworthiness and authenticity in naturalistic evaluation,” in *Naturalistic Evaluation*, ed Williams D. D. (San Francisco, CA: Jossey-Bass), 73–84.

[B45] Markus H. R. Kitayama S. (1991). Culture and self: implications for cognition, emotion, and motivation. *Psychol. Rev.* 98 224–253. 10.1037/0033-295x.98.2.224

[B46] Markus H. R. Kitayama S. (1994). “The cultural construction of self and emotion: implications for social behavior,” in *Emotion and Culture: Empirical Studies of Mutual Influence*, eds Kitayama S. Markus H. R. (Washington, DC: American Psychological Association), 89–130. 10.1037/10152-003

[B47] Markus H. R. Kitayama S. (2003). Culture, self, and the reality of the social. *Psychol. Inq.* 14 277–283. 10.1080/1047840X.2003.9682893

[B48] Markus H. R. Kitayama S. (2010). Cultures and selves: a cycle of mutual constitution. *Perspect. Psychol. Sci.* 5 420–430. 10.1177/1745691610375557 26162188

[B49] Markus H. R. Mullally P. R. Kitayama S. (1997). “Selfways: diversity in modes of cultural perception,” in *The Conceptual self in Context: Culture, Experience, Self-Understanding. The Emory Symposia in Cognition*, eds Neisser U. Jopling D. A. (New York, NY: Cambridge University Press), 13–61.

[B50] Morrow S. L. (2005). Quality and trustworthiness in qualitative research in counseling psychology. *J. Counsel. Psychol.* 52 250–260. 10.1037/0022-0167.52.2.250

[B51] Nagel J. C. (2013). *Venezuela’s Ministry of Happiness. Transitions.* Available online at: https://foreignpolicy.com/2013/10/30/venezuelas-ministry-of-happiness/ (accessed March 4, 2020).

[B52] Office for National Statistics (2019). *Measuring National well-Being in the UK: International Comparisons, 2019.* Available online at: https://www.ons.gov.uk/peoplepopulationandcommunity/wellbeing/articles/measuringnationalwellbeing/internationalcomparisons2019 (accessed March 4, 2020).

[B53] Oishi S. Graham J. Kesebir S. Galinha I. C. (2013). Concepts of happiness across time and cultures. *Pers. Soc. Psychol. Bull.* 39 559–577. 10.1177/0146167213480042 23599280

[B54] Oppong S. (2019). Overcoming obstacles to a truly global psychological theory, research, and praxis in Africa. *J. Psychol. Afr.* 29 292–300. 10.1080/14330237.2019.1647497

[B55] Osafo J. Agyapong I. Asamoah M. K. (2015). Exploring the nature of treatment regimen for mentally ill persons by neo-prophetic ministers in Ghana. *Int. J. Cult. Ment. Health* 8 325–339. 10.1080/17542863.2014.973428

[B56] Osei-Tutu A. Dzokoto V. Hanke K. Adams G. Belgrave F. (2018a). Conceptions of love in Ghana: an exploration among orthodox and charismatic Christians. *J. Psychol. Afr.* 28 83–88. 10.1080/14330237.2018.1454576

[B57] Osei-Tutu A. Dzokoto V. A. Adams G. Hanke K. Kwakye-Nuako C. Adu-Mensa F. (2018b). ‘My own house, car, my husband, and children’: meanings of success among Ghanaians. *Heliyon* 4:e00696. 10.1016/j.heliyon.2018.e00696 30094366 PMC6076215

[B58] Osei-Tutu A. Dzokoto V. A. Affram A. A. (2019). Common presenting problems in religious lay counselling practice in Ghana. *Ment. Health Relig. Cult.* 22 819–831. 10.1080/13674676.2019.1666096

[B59] Rappleye J. Komatsu H. Uchida Y. Krys K. Markus H. (2020). Better policies for better lives’? Constructive critique of the OECD’s (mis)measure of student well-being. *J. Educ. Policy* 35 258–282. 10.1080/02680939.2019.1576923

[B60] Riemer H. Shavitt S. Koo M. Markus H. R. (2014). Preferences don’t have to be personal: expanding attitude theorizing with a cross-cultural perspective. *Psychol. Rev.* 121:619. 10.1037/a0037666 25347311

[B61] Shin J. E. Suh E. M. Eom K. Kim H. S. (2018). What does “happiness” prompt in your mind? Culture, word choice, and experienced happiness. *J. Happiness Stud.* 19 649–662. 10.1007/s10902-016-9836-8

[B62] Suh E. M. Choi S. (2018). “Predictors of subjective well-being across cultures,” in *Handbook of Well-Being*, eds Diener E. Oishi S. Tay L. (Salt Lake City, UT: DEF Publishers).

[B63] Suh E. M. Oishi S. (2002). Subjective well-being across cultures. *Online Read. Psychol. Cult.* 10 10.9707/2307-0919.1076

[B64] Taylor A. (2016). *The UAE Created A Minister of Happiness, But What Does That Even Mean?* Available online at: https://www.washingtonpost.com/news/worldviews/wp/2016/02/10/the-uae-created-a-minister-of-happiness-but-what-does-that-even-mean/ (accessed March 4. 2020).

[B65] Tsai J. L. (2007). Ideal affect: cultural causes and behavioral consequences. *Perspect. Psychol. Sci.* 2 242–259. 10.1111/j.1745-6916.2007.00043.x 26151968

[B66] Tsai J. L. (2017). Ideal affect in daily life: implications for affective experience, health, and social behavior. *Curr. Opin. Psychol.* 17 118–128. 10.1016/j.copsyc.2017.07.004 28950957 PMC5659332

[B67] Uchida Y. Kitayama S. (2009). Happiness and unhappiness in east and west: themes and variations. *Emotion* 9 441–456. 10.1037/a0015634 19653765

[B68] United Nations Organization (2015). *Transforming Our World: The 2030 Agenda for Sustainable Development.* New York, NY: United Nation.

[B69] Ura K. Alkire S. Zangmo T. Wangdi K. (2012). *A Short Guide to Gross National Happiness Index: The Centre for Bhutan Studies.* Available online at: https://opendocs.ids.ac.uk/opendocs/bitstream/handle/20.500.12413/11807/Short-GNH-Index.pdf (accessed March 4, 2020).

[B70] Van der Geest S. (1998). ‘Yebisa wo fie’: growing old and building a house in the Akan culture of Ghana. *J. Cross Cult. Gerontol.* 13 333–359. 14617902 10.1023/a:1006563032706

[B71] White S. (2015). “Introduction: the many faces of wellbeing,” in *Cultures of Wellbeing: Method, Place, Policy*, eds White S. Blackmore C. (London: Palgrave Macmillan), 1–46.

[B72] White S. C. (2010). Analysing wellbeing: a framework for development practice. *Dev. Pract.* 20 158–172. 10.1080/09614520903564199

[B73] White S. C. (2017). Relational wellbeing: re-centring the politics of happiness, policy and the self. *Policy Polit.* 45 121–136. 10.1332/030557317x14866576265970

[B74] White S. C. Jha S. (2018). Towards an interdisciplinary approach to wellbeing: life histories and self-determination theory in rural Zambia. *Soc. Sci. Med.* 212 153–160. 10.1016/j.socscimed.2018.07.026 30031981

